# Chemo-mechanical behaviour of Ni-rich layered cathodes: insights from *operando* monitoring

**DOI:** 10.1093/nsr/nwae337

**Published:** 2024-09-25

**Authors:** Shiyuan Zhou, Gui-Liang Xu, Khalil Amine

**Affiliations:** Chemical Sciences and Engineering Division, Argonne National Laboratory, USA; Chemical Sciences and Engineering Division, Argonne National Laboratory, USA; Chemical Sciences and Engineering Division, Argonne National Laboratory, USA

Ni-rich layered oxides, particularly LiNi_x_Co_y_Mn_1−x−y_O_2_ (NCM, where x > 0.5) materials, have emerged as leading cathode materials for high-energy-density lithium-ion batteries (LIBs) due to their high specific capacity (>180 mAh g^–1^) and operating voltage (3.8 V vs. Li/Li^+^) [1]. Among these, polycrystalline NCM (P-NCM), composed of micron-sized secondary particles formed from nano-sized primary grain, is widely adopted in commercial applications because of its high tap density and fast lithium-ion diffusion kinetics. However, P-NCM materials face significant challenges, primarily related to capacity fading and structural degradation [2]. These issues are mainly triggered by random grain boundaries and anisotropic strain within the polycrystalline structure, particularly during high-voltage phase transitions (H_2_ → H_3_) at high states of charge (SOC). This leads to severe lattice shrinkage along the c-axis, causing electrode pulverization, surface reconstruction, and parasitic reactions. Furthermore, these problems are exacerbated as the nickel content in NCM materials rises (Ni ≥80%) and operating voltages increase (≥4.5 V), further limiting their practical applications.

To better understand these degradation mechanisms, advanced characterization techniques with improved spatial and temporal resolutions have been employed. For spatial resolution, methods such as synchrotron-based high-energy X-ray diffraction (XRD) and X-ray absorption spectroscopy have been used to obtain electrode-level crystalline structure and chemical state information [3]. Aberration-corrected scanning transmission electron microscopy has provided local atomic structures, such as distortion, grain boundary, and dislocation [4]. To further bridge the resolution gap between the electrode-level and atomic-level observations, techniques like multi-crystal rocking curve [5], full-field transmission X-ray microscopy [6], Laue diffraction microscopy [7], and Bragg coherent X-ray diffraction imaging [8] have been employed recently by offering both individual particle and statistical structural information, such as morphological, lattice strain/stress, and crystal orientation information. For temporal resolution, the transition from *ex situ* to *in situ* and *operando* measurement techniques has significantly advanced the field [9]. *In situ* techniques focus on key working conditions such as the electrolyte environment and electric field, while *operando* techniques monitor processes related to the real electrochemical behaviours, especially in practical configurations like coin-type or pouch cells. These approaches offer critical advantages, including minimal disruption to the electrochemical process and real-time tracking of structural changes, making them essential for tracking thermodynamically unstable products and studying non-equilibrium reactions. Ultimately, the integration of both spatial and temporal resolution techniques under realistic working conditions is pivotal for gaining deeper insights into the chemo-mechanical behaviours of NCM cathodes and improving LIBs performance.

**Figure 1.**
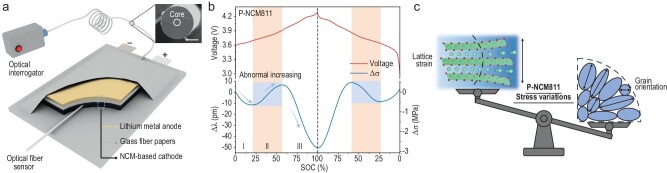
(a) Schematic illustration of *operando* FBG sensors embedded in a NCM-based pouch cell. (b) *Operando* monitoring of stress variation of P-NCM cathode materials. (c) Balance between lattice strain and grain orientation in alleviating P-NCM stress variations. Fig. 1a and b were reproduced from ref. [10] with permission.

Huang *et al*. recently introduced an *operando* technique using Fiber Bragg Grating (FBG) sensors to monitor stress evolution in P-NCM cathodes during battery cycling in a pouch cell, as shown in Fig. 1a [10]. FBG sensors detect stress by measuring shifts in the Bragg wavelength, offering significant advantages due to their small size, flexibility, and non-invasive nature. By embedding the FBG sensor directly into the P-NCM cathode and roll-pressing the electrodes to ensure tight contact, the study was able to capture stress variations in real-time, driven by the expansion and contraction of accumulated NCM particles, ideally at the material level. This instrumental advancement led to the discovery of an abnormal stress variation (Δσ) in P-NCM cathodes (Fig. 1b). The stress exhibited a non-monotonic behaviour, with three distinct regions observed during both charging and discharging cycles. While stress typically decreased during charging and increased during discharging, an unexpected rise in stress was noted during specific phases of lithiation (Region II). In contrast, single-crystal NCM (S-NCM) displayed a more predictable, monotonic stress evolution throughout the cycling process, with no abnormal stress peaks. Using XRD and electron backscatter diffraction (EBSD) techniques, the authors investigated the causes of the stress anomalies. They found that the abnormal stress behaviours in P-NCM cathodes was caused by the anisotropy of primary particles within the polycrystalline structure. During de-lithiation, individual grains within the P-NCM material expanded and contracted unevenly due to their varied orientations, leading to the formation of internal microcracks. These microcracks disrupted the normal stress response, degraded the structural integrity, and increased the surface area for parasitic reactions with the electrolyte, accelerating capacity loss. In contrast, S-NCM, with its single-crystal structure, experienced minimal internal stress variation and demonstrated superior cycling stability. To further reduce structural stress in polycrystalline materials, the authors modified the arrangement of primary particles, synthesizing Gd-doped P-NCM materials with an ordered grain structure. These Gd-doped P-NCM cathode materials exhibited reduced stress anomalies and improved cycling stability, achieving a capacity retention of 82% after 500 cycles.

In summary, by utilizing an *operando* FBG sensor, Huang and co-workers captured real-time stress variations in P-NCM materials, revealing an abnormal stress pattern linked primarily to the orientation of primary particles rather than chemical strain (Fig. 1c). This discovery provides critical insights into the mechanical degradation mechanisms of P-NCM cathodes. Looking ahead, this work highlights several strategies for optimizing the performance of Ni-rich layered oxide cathodes. One approach is to reduce the anisotropy of polycrystalline particles by controlling grain orientation, thereby mitigating stress-related degradation. Another potential solution is to transition entirely to single-crystal materials, which naturally exhibit less mechanical stress. From the perspective of advanced characterization techniques, the relationships between particle morphology, chemical states, reaction homogeneity/heterogeneity, and overall cell performance are complex, with influences spanning multiple length and time scales. Thus, continued advancements in these characterization methods are crucial for further understanding and enhancing the performance of next-generation LIBs.
